# Prevalence of Dietary Supplements Use among Gymnasium Users

**DOI:** 10.1155/2017/9219361

**Published:** 2017-04-05

**Authors:** Ayman H. Jawadi, Abdulmalik M. Addar, Abdulaziz S. Alazzam, Fahad O. Alrabieah, Abdullah S. Al Alsheikh, Roaa R. Amer, Al Anoud S. Aldrees, Maha A. Al Turki, Ali K. Osman, Motasim Badri

**Affiliations:** ^1^College of Applied Medical Sciences, KSAU-HS, Riyadh, Saudi Arabia; ^2^College of Medicine, KSAU-HS, Riyadh, Saudi Arabia; ^3^College of Public Health and Health Information, KSAU-HS, Riyadh, Saudi Arabia

## Abstract

*Background.* Several studies showed that regular gymnasium users use various dietary supplements without comprehension of their potential risks.* Objective.* To determine the prevalence and dietary supplement intake and assess the awareness of supplement use among regular gymnasium users in Riyadh, Saudi Arabia.* Methods.* A descriptive cross-sectional study was conducted among regular gymnasium users in Riyadh, Saudi Arabia, between April 2015 and June 2015. A validated structured questionnaire was used.* Results.* The study included 299 participants. Of these 113 (37.8%) were dietary supplements users and this was more common among males than females (44.7% versus 16.4%). Gender based analysis showed that males were exercising more frequently than females and the type of cardiovascular exercise was more among them. The most commonly used supplements were whey protein (22.1%), amino acids (16.8%), multivitamins (16.8%), creatine (11.5%), and omega 3 (11.5%). The reasons for taking dietary supplements were to improve body shape (47.7%), increase health (44.2%), and improve performance (41.5%).* Conclusion.* Most of the information about supplements was obtained from unreliable sources. More studies are needed to better understand supplements use and their impact on health in Saudi Arabia.

## 1. Introduction

The Kingdom of Saudi Arabia has an expanding population in which the young constitute the majority, with an increasing number of people attending athletic activities and easy access to dietary supplements [1]. The Dietary Supplement Health and Education Act defines dietary supplements as “any product (other than tobacco) intended to supplement the diet that contains one or more dietary ingredients.” They embrace diet fortifying nutritional elements such as herbs, vitamins, meal supplements, and nutritional athletic merchandise midst others [2–4]. According to the United States of America Food and Drug Administration (FDA) any product labeled as a “supplement” means that its contents and the claims on the label have not been approved or evaluated by the FDA [5, 6]. It is well documented that the use of some of these products may lead to serious health injury [7]. The regular gymnasium users are at risk resulting from taking various categories of nutritional supplements intended for athletic improvement [8].

Popularity of these supplements along with their easy access makes us question their consumption among regular gymnasium users, who engage in sports for pleasure and not monetary gain or professional benefits, in Saudi Arabia. There was only one research discussing supplement use among professional athletic football players in Saudi Arabia that included 105 athletes of whom 93.3% were using dietary supplements, that is, sports drinks 88.7%, vitamin C 82.6%, multivitamins 52%, omega 6 18.6%, creatine 16.3%, and Ginkgo biloba 10.2%. The majority of participants reported improvement of health and performance as the main reason for using dietary supplements [2]. However, there is no data addressing this issue in nonprofessional athlete in Saudi Arabia. In Beirut, Lebanon, 36.3% of gymnasium users were reported to use supplements [9]. Dietary supplements are used for different intentions such as building muscles, better health, and improving performance. Some studies have shown that people have different opinions about the use of dietary supplements [10]. Supplement users have different sources of information about the supplements they are taking. Studies from Lebanon and Iran reported that coaches are the main source of information about dietary supplements among users [9, 11].

This research is carried out to shed light on nonprofessional use of dietary supplements among both genders in Saudi Arabia. Since there is no data available regarding this issue of the regular gym exercises supplement use, it is crucial to address this important topic.

## 2. Methods

A descriptive cross-sectional study was conducted in the city of Riyadh, Saudi Arabia, from April 2015 to June 2015. A pilot study was conducted and showed that 17/51 = 33.3% of the respondents were using supplements. Therefore, at 80% power and 95% confidence level, the required sample size was calculated to be 342~350 participants. However, due to the difficulty of participant recruitment only 299 participants' responses were collected. This is mainly due to difficulty faced in getting consent of female health centers. Culturally it is not acceptable to disturb or break trainer's confidentiality. Thus, the major health centers in Riyadh were identified and those ones who were willing to participate in the study were visited. The sampling technique used was purposive sampling. Subjects interviewed had to be gymnasium users, who engage in sports for pleasure and not monetary gain or professional benefits. Both males and females aged between 18 and 45 years being in Riyadh at the time of the study were included. Professional athletes (a person who takes sports as their main monetary gain), coaches, abusive drug users, and participants below 18 years of age or over 45 years were all excluded. All participants signed a formal consent after being informed about the objectives of the study. The Ethics Committee of King Abdullah International Medical Research Centre approved the study.

An individual interview questionnaire which was previously tested in a pilot study and final version was used to collect information about demographic and socioeconomic data, lifestyle, type of supplements, measuring blood pressure, frequency, sources of information, and beliefs regarding supplements use. Finally, anthropometric parameters weight (Kg) and heights (Cm) were taken and then Body Mass Index (BMI) was calculated to assess physical status. Anthropometric measurements and demographic data were collected to explore the possibility of association between the consumption of dietary supplements and these parameters. All questions and measurements were taken by the researchers themselves. Blood pressure measurement was done using a British hypertension society certified blood pressure machine, Omron M7 (HEM-780-E). Weight and height were measured using equipment available at the visited health clubs.

Data were summarized medians (IQR) or proportions and compared using the Mann–Whitney or *χ*^2^ square test. Statistical analysis was conducted using the Statistical Package for Social Studies software (IBM SPSS version 19; USA). All tests were two-sided and a *P* value <0.05 was considered significant.

## 3. Results

The study included 299 participants. The prevalence of dietary supplements use among the gymnasium users was 113/299 (37.8%).  Table 1 shows the demographic characteristics of supplement users and nonusers. Age did not differ between the two comparison groups (*P* = 0.133). Gender distribution was significantly different in the two groups (*P* < 0.0001) with males amounting to 89.4% of dietary supplements users and 67.2% of nonusers, and female dietary supplements users and nonusers constituted 10.6% and 32.8% of study participants, respectively. Saudi nationals predominate among study participants; 82.3% and 78.8% of dietary supplements users and nonusers were Saudis. However no significant difference was observed between the two groups in terms of nationality (*P* = 0.187). Smoking status differed significantly between the two groups (*P* = 0.005); smokers amounted to 45.1% of dietary supplements users versus 29% of non-supplements users. Level of education was not significantly different between the two groups (*P* = 0.945). Those with bachelor degree were the group with highest frequency among both dietary supplements users (54.9%) and nonusers (58.1%) compared with participants with other levels of education.

Level of income was approximately similar in the two groups (*P* = 0.157). The highest percentage of participants among dietary supplements users and nonusers was those with lowest income: 34.5% and 31.2% with income within 3,000 < 5,000 SR (1 USD = 3.75 SR), followed by 29.2% and 28.5% with income within 10,000 < 20,000 SR, respectively.

Gender based analysis was done and showed that males were exercising more frequently than females and for a longer period. No difference was found in smoking status, type of supplements, sources of information, type of exercise, and beliefs between male and female participants.

The most used supplements users acquired information mainly from online sources (38%) followed by advice from coach (35.4%), by physician (13.3%), academic journals (12.4%), dietician (11.5%), and magazines (3.5%) (Table 2). No statistically significant difference was found between male and female gymnasium users for source of information about supplement use.

Height (in meter) among the supplement users and nonusers was 1.73 and 1.70 while their weight (kg) was 74.6 and 79, respectively (Table 3). The BMI among supplement users and nonusers was 24.8 and 27.6, respectively, indicating that the non-supplement users were overweight as compared with the users and the difference was statistically significantly (*P* < 0.0001). This observation could be due to the fact that supplement users were exercising for a longer time (<10 years) than nonusers (Table 4).

The diastolic blood pressure median was significantly higher (*P* < 0.0001) among the non-supplement users as compared with the users (72 versus 77 mmHg). Although this difference was statistically significant it is within normal range. The systolic blood pressure among the two groups was 119 versus 121, respectively, but the difference was not statistically different. Differences between males and females are also shown. Compared with females, males were significantly taller (*P* < 0.0001), were more obese (*P* < 0.0001), and had lower median diastolic blood pressure (*P* < 0.0001) (Table 3).

Duration of exercise for less than one year was highest among nonusers of supplements (61.8% versus 26.5%) and males were exercising for a longer time than females (*P* < 0.000) (Table 4). The frequency of exercise was highest among those who exercise 3–5 days per week and was higher among supplement users than nonusers (66.4% versus 54.8%). Approximately 50% versus 12% of the supplement users and nonusers do cardiovascular exercise and males were exercising more frequently than females, which was statistically significant (*P* < 0.000).

In terms of type of exercise, supplement users were mainly practicing cardiovascular training while nonusers were doing other types of exercise more frequently (49.6% versus 60.8; *P* < 0.0001). Female gymnasium users were practicing into other types of exercise than males (72.6% versus 47.8%) (*P* < 0.000). Length of sleep did not differ significantly between the two groups (*P* < 0.372). However, males were getting less sleep than females (*P* < 0.006). The major reported reasons for supplements use were for appearance (47.7%) and to improve health (44.2%) and performance (41.5%) (Table 5). Others were to lose weight (11.5%), to prevent injury (8.8%), and for recovery (6.1%). No statistically significant difference was found between male and female gymnasium users for the reason of using supplements.

The most common belief among all participants was that supplements increase the amount of training that the participant can undergo, followed by the belief that supplements provide more energy and make the participants healthier (Table 6).

## 4. Discussion

Supplements are increasing in use in Saudi Arabia and becoming easier to acquire. They are tempting to use to get better exercise outcomes. With the potential harm these supplements may have, it is important to address their availability among regular gymnasium users. No difference was found between genders in smoking status, type of supplements, sources of information, type of exercise, and beliefs and for this reason comparisons were not made between male and female supplements users. A study conducted in Finland Olympic athletes concluded that of 81% of those who use nutritional supplements only 27% of them had the opportunity to consult a dietary specialist before using them [12]. Another study done in the United States showed that 89% of adult athletes were using at least one supplement at the time of the study [13]. A study conducted in New York in 2004 found the prevalence of supplements to be 84.7% among male athletes [14]. Another study conducted in Lebanon in 2012 reported that the prevalence of supplements use was at 36.3% among exercisers in Beirut, Lebanon, gyms [9]. Supplements use among regular gymnasium users in Saudi Arabia was not sufficiently investigated. There was only one study in Saudi Arabia that has investigated supplements use among professional soccer players [2].

Due to the lack of data about supplements use among regular gymnasium users in Saudi Arabia, we conducted this study to try to understand the pattern of use among this population and to explore beliefs and intentions regarding supplements use. The Lebanese study was very comparable to our study and used similar methods. Our study found supplements use to be at 37.8% versus 36.3% in Lebanon. This might be due to similarity of age distribution of participants of the two studies, as most of our study population was between 22 and 33 versus 20 and 30 years old in the Lebanese study. Our findings showed that males form 89.4% and females 10.6% of the participants. In addition, sex difference between users and nonusers was statistically significant and users were more common among males. This finding concurs with the Lebanese study where males constituted 72% of supplements users while females amounted to 28%. In Isfahan, Iran, males constituted 86.8% of study participants and females 11.2% [9, 11]. This can be attributed to the fact that males were exercising more frequently than females. Both our population and the Lebanese showed that most of the subjects fall in the bachelors' degree educational status [9]. In contrast to the Lebanese study, we found a significant difference between the smoking status of supplements users and nonusers with smokers constituting 45.1% of users and 29% of nonusers [9].

Respondents consume a total of 25 supplements with the most common used supplements being whey protein (22.1%), amino acids (16.8%), multivitamins (16.8%), creatine (11.5%), and omega 3 (11.5%) while the Lebanese study showed almost similar results with protein powder (39.8%), amino acid pills (34.9%), whey protein (32.3%), creatine (19.4%), and multivitamins (17.7%) [9]. In comparison to male athletes in New York, the most common used supplement is multivitamin/minerals (45%), followed by protein shakes/bars (42.3%), vitamin C (34.7%), and vitamin E (23.4%) [7]. It is reported that some supplements that athletes use may cause some harm. For example, the amino acid has been shown to cause gastrointestinal adverse effects, mainly diarrhea and stomach cramps [15]. Creatine supplementation has short- and long-term side effects. The side effects of short-term use for 3 to 5 days include an increase in body weight due to increase in total body water [16], and the long-term use of creatine (2 months) may result in limb edema in some trainers [17]. Despite these minimal side effects, the supplements mentioned above are legal to use by the International Olympic Committee (IOC) and the National Collegiate Athletic Association (NCAA) [18, 19]. No statistical difference between the frequency of exercise between users and nonusers was found in our study, a finding in agreement with the Lebanese study [9].

We found that most of the information pertaining to supplements use was obtained from unreliable sources such as online sources (38%) and coaches (35.4%). Physicians (13.3%), academic journals (12.4%), and dieticians (11.5%) were less common as a source of information. The Lebanese study similarly reported coaches (44.6%) and online sources (36.6%) were the major source of information [9]. The study in Isfahan, Iran, reported coaches (65%), nutritionists (30%), and doctors (25%) were the main sources of information to supplement users [11]. Saudi professional athletes reported that more reliable information sources were obtained from physicians (45.9%) and dieticians (28.5%) [2].

Furthermore, we investigated reasons for using supplements to understand why regular gymnasium users take them. Our subjects reported that the main reason is for better appearance (47.7%), to improve health (44.2%), and to improve performance (41.5%). On the other hand, the main reasons for their use among professional athletes in Saudi Arabia were to improve performance (43.8%) and to improve health (32.6%) [2]. It is surprising that both professional athletes (43.8%) and regular gymnasium users (41.5%) respond similarly for using supplements to improve performance [2]. The New York study assessed the same issue and the results were comparable to ours. The most common reasons were building muscles (49.1%), preventing future illness (38.4%), increasing energy (36.1%), improving performance in sports (24.4%), gaining strength (22.4%), and aiding in recuperation (20.5%) [14].

Cardiovascular exercise was most common (49.6%) among supplement users of our study participants. This is in contradiction to the Lebanese study where most supplement users (80.6%) were engaged in strength training [9]. A statistically significant difference (*P* < 0.0001) in our study was found between supplement users and nonusers in terms of type of exercise practiced. Most supplement users were exercising for more than 1 year (75.2%) and most nonusers exercised for less than 1 year (61.8%), meaning that the more time spent exercising increases the likelihood of using supplements. Median BMI for supplements users was 24.8 and nonusers 27.6, meaning that nonusers were significantly overweight in comparison with users (*P* < 0.0001). Diastolic blood pressure was significantly lower for users (72) compared with nonusers (77) (*P* < 0.0001) but it is still within the normal range. From the anthropometric measurements, we can deduce that users are in better physical shape than nonusers. This might be due to the longer period of exercise as most supplement users were exercising for more than 10 years and are more motivated. Although statistically significant, this might not necessarily conclude a strong correlation between the physical status and supplement use as it should be explored more on a larger sample of participants. No difference was found between supplement users and nonusers in relation to educational status, income, and sleeping pattern.

Our study is not without limitations, several of which should be acknowledged. We had difficulties in recruiting female participants. This resulted in most of our study participants being male. This is due to difficulty faced in getting approval by female gymnasia and this is due to cultural restrictions and not to disturb gymnasium users or break their confidentiality. We had some difficulties in identifying which supplements were common to be included in our questionnaire but we included a blank space to write which supplements the respondent was taking. Future studies should use sampling methods similar to other studies to make comparisons feasible. We recommend future studies to include a larger sample size with more female participants and use a standard data collection method.

In conclusion, supplement use among regular gymnasium users in Riyadh is common and more dominant among males. The main reasons for supplements use among our study participants were for better appearance and to improve health and performance. More studies are needed to have a more detailed depiction regarding supplements use among regular gymnasium users in Saudi Arabia. Health professionals including physicians and nutritionists need to be available in gymnasium centers to provide reliable information regarding supplements and their health hazards. In addition, awareness programs that explain the potential side effects of supplements and their interactions and reliable information sources should be made available.

## Figures and Tables

**Table 1:** Demographic characteristics of participants.

Characteristic	Supplements users *n* (%)	Non-supplements user *n* (%)	*P* value
Age (years), median (IQR)	27 (22–31)	28 (23–33)	0.133
Sex			
(i) Male	101 (89.4)	125 (67.2)	<0.0001
(ii) Female	12 (10.6)	61 (32.8)	
Smoking status			
(i) Yes	51 (45.1)	54 (29)	0.005
(ii) No	62 (54.9)	132 (71)	
Nationality			
(i) Saudi	93 (82.3)	141 (78.8)	0.187
(ii) Non-Saudi	20 (17.7)	45 (24.2)	
Level of education			
(i) Less than high school	1 (0.9)	2 (1.1)	0.945
(ii) High school	35 (31)	52 (28)	
(iii) Bachelor	62 (54.9)	108 (58.1)	
(iv) Master/Ph.D.	15 (13.3)	24 (12.9)	
Income (SR)			
(i) 3,000 to <5,000	39 (34.5)	58 (31.2)	0.157
(ii) 5,000 to <10,000	29 (25.7)	35 (18.8)	
(iii) 10,000 to <20,000	33 (29.2)	53 (28.5)	
(iv) 20,000 to <30,000	8 (7.1)	25 (13.4)	
(v) >30,000	4 (3.5)	15 (8.1)	

*P* value: Mann–Whitney for difference in medians and *χ*^2^ test for difference proportions.

**Table 2:** Source of information.

Source of information	*n* (%)	Males (%)	Females (%)	*P* value
Online	43 (38)	34 (15)	9 (12.3)	0.565
Coach	40 (35.4)	29 (12.8)	11 (15.1)	0.626
Physician	15 (13.3)	10 (4.4)	5 (6.8)	0.409
Academic journals	14 (12.4)	12 (5.3)	2 (2.7)	0.366
Dietician	13 (11.5)	11 (4.9)	2 (2.7)	0.438
Magazines	4 (3.5)	4 (1.8)	0 (0)	0.252

*P* value: *χ*^2^ test.

**Table 3:** Anthropometric measurement and blood pressure.

Characteristic	Supplements users	Non-supplements user	*P* value	Males	Females	*P* value
Height (meters), median (IQR)	1.73 (1.67–1.76)	1.70 (1.64–1.76)	0.021	1.73 (1.69–1.77)	1.64 (1.59–1.68)	0.000
Weight (KGs), median (IQR)	74.6 (68–82.8)	79 (69.4–89.3)	0.010	79.5 (71.5–89.2)	68 (62–77.5)	0.000
Body Mass Index, median (IQR)	24.8 (23.3–27.5)	27.6 (24.7–30.3)	<0.0001	26.4 (24.2–29.7)	26 (22.8–28.5)	0.050
Diastolic, median (IQR)	72 (65.5–79)	77 (70–85)	<0.0001	74 (67–80)	80 (70–89)	0.000
Systolic, median (IQR)	119 (111–128)	121 (113–128)	0.283	121 (113–128)	120 (110–128)	0.073

*P* value: *χ*^2^ test.

**Table 4:** Duration and type of exercise.

Characteristic	Supplements users *n* (%)	Non-supplements users *n* (%)	*P* value	Males (%)	Females (%)	*P* value
*Duration of exercise (years)*						
<1	30 (26.5)	115 (61.8)	<0.0001	90 (39.8)	55 (75.3)	0.000
1–5	57 (50.4)	51 (27.4)		93 (41.2)	15 (20.5)	
>5−10	16 (14.2)	14 (7.5)		28 (12.4)	2 (2.7)	
>10	10 (8.8)	6 (3.2)		15 (6.6)	1 (1.4)	
*Frequency of exercise (days per week)*						
1-2	4 (3.5)	24 (12.9)	0.016	6 (2.7)	22 (30.1)	0.000
3–5	75 (66.4)	102 (54.8)		142 (62.8)	35 (47.9)	
>5	34 (30.1)	60 (32.3)		78 (34.5)	16 (21.9)	
*Types of exercise*						
Cardiovascular	56 (49.6)	22 (11.8)	<0.0001	72 (31.9)	6 (8.2)	0.000
Weight training	8 (7.1)	47 (25.3)		43 (19)	12 (16.4)	
Mixed	1 (0.9)	4 (2.2)		3 (1.3)	2 (2.7)	
Other	48 (42.5)	113 (60.8)		108 (47.8)	53 (72.6)	
*Length of sleep (hours)*						
<6	28 (24.8)	33 (17.7)	0.372	50 (22.1)	11 (15.1)	0.006
6–8	70 (61.9)	133 (71.5)		156 (69)	47 (64.4)	
>8–10	13 (11.5)	18 (9.7)		16 (7.1)	15 (20.5)	
>10	2 (1.8)	2 (1.1)		4 (1.8)	0 (0)	

*P* value: *χ*^2^ test.

**Table 5:** Reasons for supplement use.

Reason of supplement use	Males (%)	Females (%)	*N* (%)	*P* value
Appearance	42 (18.6)	12 (16.4)	54 (47.7)	0.679
Improve health	36 (15.9)	14 (19.2)	50 (44.2)	0.518
Improve performance	38 (16.8)	9 (12.3)	47 (41.5)	0.360
Lose weight	10 (4.4)	3 (4.1)	13 (11.5)	0.909
Prevent injury	5 (2.2)	5 (6.8)	10 (8.8)	0.055
Recovery	3 (1.3)	4 (5.5)	7 (6.1)	0.041

*P* value: *χ*^2^ test.

**Table 6:** Beliefs about supplements use.

Belief about supplements	Group	*n* (%)	Agree	Somewhat agree	Somewhat disagree	Disagree	Do not know
Dietary supplements make me healthier	Users	*n*	46	39	1	26	1
		%	40.7	34.5	0.9	23	0.9
	Nonusers	*n*	44	46	17	67	12
		%	23.7	24.7	9.1	36	6.5
*P* value	0.000						

Dietary supplements are safe to use	Users	*n*	41	41	6	20	5
		%	36.3	36.3	5.3	17.7	4.4
	Nonusers	*n*	31	56	21	62	16
		%	16.7	30.1	11.3	33.3	8.6
*P* value	0.000						

Dietary supplements increase the amount of training I can undergo	Users	*n*	67	18	2	21	5
		%	59.3	15.9	1.8	18.6	4.4
	Nonusers	*n*	78	40	17	35	16
		%	41.9	21.5	9.1	18.8	8.6
*P* value	0.010						

Dietary supplements provide me with more energy	Users	*n*	51	24	6	27	5
		%	45.1	21.2	5.3	23.9	4.4
	Nonusers	*n*	68	23.7	15	38	21
		%	36.6	15	8.1	20.4	11.3
*P* value	0.169						

Dietary supplements increase my ability to cope with pain	Users	*n*	23	19	8	48	15
		%	20.4	16.8	7.1	42.5	13.3
	Nonusers	*n*	30	22	21	58	55
		%	16.1	11.3	11.3	31.2	29.6
*P* value	0.008						

Dietary supplements improve my concentration	Users	*n*	33	20	6	42	12
		%	29.2	17.7	5.3	37.2	10.6
	Nonusers	*n*	28	33	16	60	49
		%	15.1	17.7	8.6	32.3	26.3
*P* value	0.002						

*P* value: *χ*^2^ test.
